# Rare But Specific B‐Cell Receptor Reactivity Against Coxsackievirus A24 in Primary Central Nervous System Lymphomas

**DOI:** 10.1002/jha2.70422

**Published:** 2026-10-08

**Authors:** Igor Age Kos, Onur Cetin, Natalie Fadle, Evi Regitz, Klaus‐Dieter Preuss, Stefan Lohse, Sigrun Smola, Sylvia Hartmann, Falko Fend, Karin Klingel, Florian Scherer, Elisabeth Schorb, Moritz Bewarder, Philipp Staber, Lorenz Thurner

**Affiliations:** ^1^ José Carreras Center for Immuno‐ and Gene Therapy of Department of Internal Medicine I, Hematology, Oncology, Rheumatology and Clinical Immunology Saarland University Medical School Homburg Germany; ^2^ Institute of Virology Saarland University Medical Center Homburg Germany; ^3^ Helmholtz Institute for Pharmaceutical Research Saarland (HIPS) Helmholtz Centre for Infection Research Saarbrücken Germany; ^4^ Institute of Pathology University Hospital Essen Essen Germany; ^5^ Institute of Pathology and Neuropathology University Hospital Tübingen Tübingen Germany; ^6^ Department of Medicine I Medical Center Faculty of Medicine University of Freiburg Freiburg Germany; ^7^ German Cancer Consortium (DKTK) Partner Site Freiburg and German Cancer Research Center (DKFZ) Heidelberg Germany

## Abstract

**Introduction:**

Primary central nervous system lymphoma (PCNSL) remains a distinct and aggressive malignancy whose underlying antigenic triggers are largely unknown. While autoantigens such as neurabin‐I and SAMD14 have been implicated, the potential role of infectious agents in driving clonal B‐cell receptor selection within the central nervous system remains less understood.

**Methods:**

A viral proteome microarray screening approach encompassing 158 viral entities was utilized to test tumor‐derived recombinant B‐cell receptors from PCNSL. Furthermore, we analyzed a validation cohort comprising 22 PCNSL patients and tested expanded control sera from 188 healthy individuals. Functional assays were performed to confirm B‐cell receptor activation and downstream signaling upon cognate antigen engagement.

**Results:**

Screening identified a distinct B‐cell receptor reactivity directed against Coxsackievirus A24 in a PCNSL case, which was completely absent across non‐PCNSL lymphomas. Validation testing in an independent cohort confirmed rare reactivity (1/22, 4.55%). Control serum analysis revealed weak reactivity in 1 out of 188 healthy individuals (0.53%). Functional assays demonstrated that the Coxsackievirus A24‐derived antigen is capable of triggering antigen‐specific receptor signaling.

**Conclusion:**

We report the first evidence linking PCNSL B‐cell receptor reactivity to a viral antigen, identifying Coxsackievirus A24 as a rare ligand. While functional assays confirm the capacity for receptor activation, the findings must be interpreted cautiously as hypothesis‐generating. Further investigation in larger independent cohorts is required to determine whether viral antigens play any meaningful pathogenic role in this malignancy.

**Trial Registration:**

The authors have confirmed clinical trial registration is not needed for this submission.

Dear Editor,

Primary central nervous system lymphoma (PCNSL) represents a specific extranodal subtype of diffuse‐large B‐cell lymphoma (DLBCL) with exclusive manifestation in the CNS. It presents frequently with high similarities to MCD type or Cluster 5 (C5), often with mutations in MYD88 and CD79B, and overrepresentation of IGHV4‐34 [1, 2]. Even before these molecular alterations of the B‐cell receptor (BCR) pathway were identified, it has been suggested that BCR stimulation by specific antigens might play a pathogenic role. Previously, we have identified SAMD14/neurabin‐I as target antigens of PCNSL BCRs, with the peculiarity of immunogenic hyper‐N‐glycosylations of ASN 339 of SAMD14 and ASN 1277 of neurabin‐I [3]. SAMD14 is a sterile α‐motif‐containing protein implicated in hematopoietic progenitor signaling, whereas neurabin‐I is a neural tissue‐enriched F‐actin‐ and protein phosphatase‐1‐binding scaffold involved in cytoskeletal organization and neuronal signaling. SAMD14 and neurabin‐I share a highly homologous sterile α‐motif domain. Reactivity against these proteins was found in 8 of 12 PCNSL‐derived BCRs (67%). Antigen engagement also induced BCR pathway activation and proliferation in experimental lymphoma models, supporting a potential role for chronic autoantigenic stimulation in a subgroup of PCNSL. Similarly, for primary intraocular lymphoma (PIOL) hyper‐N‐glycosylated SEL1L3 was identified as an atypically post translationally modified target autoantigen of the BCR [4]. In systemic DLBCL post translational modifications also seem to play a role in BCR reactivity and lymphomagenesis [5].

In contrast to relatively well‐studied genetic or epigenetic pathway alterations, little is known about specific and complementary external stimuli of different subgroups of DLBCL [5, 6, 7, 8]. For several lymphoma entities infectious external triggers have been identified. For example, antigens of hepatitis C virus in splenic marginal zone lymphoma (SMZL), or *Helicobacter pylori* in gastric extranodal marginal zone lymphoma, or *Moraxella catarrhalis* RpoC together with MID/Hag, or *Rothia muciliaginosa* in NLPHL. Eradication of the infectious trigger belongs to the standard therapy for MZL [9, 10, 11]. In PCNSL, however, evidence for infectious triggers remains limited. The best‐established viral association is Epstein–Barr virus in immunodeficiency‐associated PCNSL, whereas EBV is absent or detected only rarely in PCNSL arising in immunocompetent patients. Beyond EBV, no reproducible infectious trigger has been established in PCNSL. This limited evidence provided the rationale for the present unbiased screening of PCNSL BCRs against viral antigens.

Here we report our findings in a retrospective study that aimed to screen for specific reactivities of PCNSL‐BCRs against infectious antigens.

## 1 Methods and Results

Recombinant PCNSL‐Fabs of 15 patients with PCNSL were screened at concentrations of 1, 10, and 20 µg/mL on a custom‐made VIROME microarray representing 2206 viral genes chosen from 158 viruses using nucleic acid programmable protein array technology (ASU, Biodesign Institute, Arizona State University, USA). Screening and analysis of the arrays were performed by ASU, which showed reactivities to several candidate antigens (Figure 1A). The pool producing a reproducible signal against the Coxsackievirus A24‐derived clone was subsequently deconvoluted by individual testing of its constituent Fabs, identifying one reactive Fab among the 15 PCNSL Fabs subsequent dot‐blot analysis and ELISA verified Coxsackievirus A24 antigen clone CaCD00959607 as a specific target.

**FIGURE 1:**
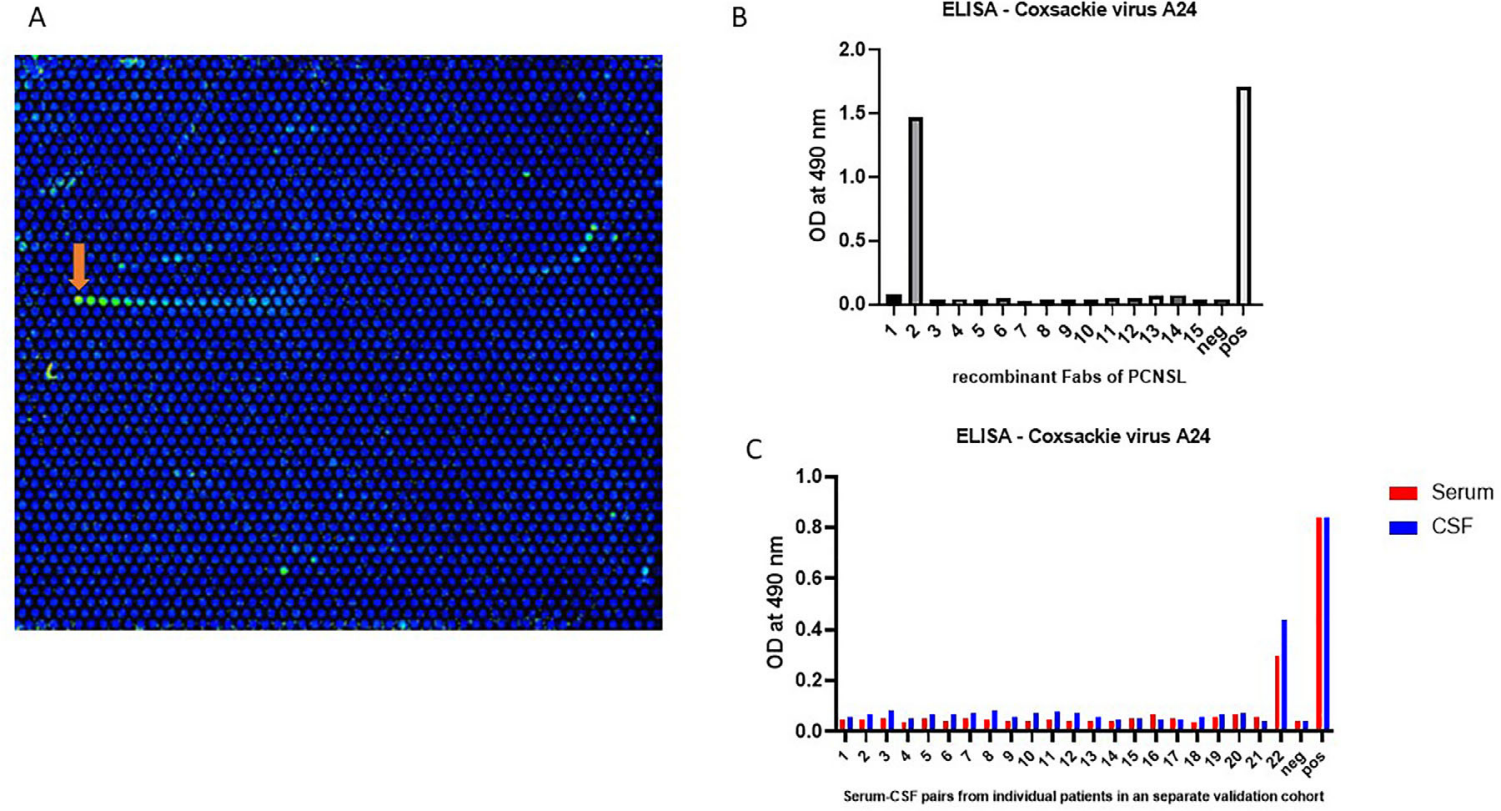
Identification and validation of B‐cell receptor reactivity against a Coxsackievirus A24‐derived antigen in PCNSL. (A) Representative VIROME protein microarray showing candidate antigen reactivities of recombinant PCNSL‐derived Fabs. The arrow indicates reactivity against the Coxsackievirus A24‐derived antigen clone CaCD00959607. Additional signals along the same horizontal line were attributed to streaking on the array. Coxsackievirus A24 reactivity was subsequently evaluated by ELISA. (B) ELISA of recombinant Fabs derived from 15 patients with PCNSL against the Coxsackievirus A24‐derived antigen CaCD00959607. One of the 15 PCNSL‐derived Fabs showed marked reactivity. Pos: positive control, Neg: negative control. (C) ELISA analysis of paired serum and cerebrospinal fluid (CSF) samples from 22 patients with PCNSL in an independent validation cohort. Reactivity against the Coxsackievirus A24‐derived antigen was detected in paired serum and CSF samples from one patient. Optical density (OD) was measured at 490 nm.

Of note, recombinant Fabs of 153 patients with several other entities, including lymphoplasmacytic lymphoma, mantle cell lymphoma, angioimmunoblastic T‐cell lymphoma, primary vitreoretinal lymphoma, Burkitt lymphoma, nodular lymphocyte predominant Hodgkin lymphoma, cases of classic Hodgkin lymphoma with BCR, follicular lymphoma and DLBCL were also tested against the Coxsackie A24 antigen (Supporting Information). Among the lymphoma entities, reactivity was only observed in the PCNSL samples. None of the other investigated viral antigens demonstrated any relevant reactivity with PCNSL (Figure 1B). This recombinant PCNSL‐Fab was not reactive against neurabin‐I/SAMD14. In serum/cerebrospinal fluid (CSF) paired samples of a different validation cohort of 22 patients with PCNSL, reactivities against Coxsackie A24 antigen clone CaCD00959607 were found in 1/22 patients both in CSF and plasma (Figure 1C), again in this case also no reactivity against SAMD14/neurabin‐I had been found before. The antibodies had a titer of 1:400 in the plasma and 1:50 in CSF and were of IgG class. Since most seroprevalence studies of Coxsackievirus A24 were conducted in post‐endemic settings, we investigated the serological status of 188 healthy donors using the same methodology. Serum from one donor was found to be weakly reactive to the target antigen, showing a prevalence of 0.53% versus 4.55% in the PCNSL cohort.

Cryo‐preserved lymphoma samples of the patient showing reactivity to the coxsackie A24v strain and two other controls were tested for viral RNA. We found no traces of coxsackie A24v RNA in the tissue.

To assess the functional relevance of Coxsackievirus A24 antigen recognition, we used a previously established doxycycline‐inducible BCR expression system in the ABC‐DLBCL cell lines TMD8 and U2932, as described previously [3]. Because no suitable EBV‐negative PCNSL cell line is available, the cells were engineered to express either the Coxsackievirus A24‐reactive PCNSL BCR or a SAMD14/neurabin‐I‐reactive PCNSL BCR as an antigen‐specific control. Following doxycycline induction, cells were stimulated with the recombinant Coxsackievirus A24‐derived antigen CaCD00959607 or SAMD14 at 5 µg/mL, with anti‐IgM at 1 µg/mL serving as a positive control. BCR pathway activation was assessed by immunoblotting for phosphorylated SYK, BTK, PLCγ2, and BLNK, as well as c‐MYC expression. Actin was used as a loading control, and non‐induced cells were included to assess dependence on recombinant BCR expression.

Stimulation with the Coxsackievirus A24‐derived antigen induced phosphorylation of SYK, BTK, PLCγ2, and BLNK and increased c‐MYC expression in TMD8 and U2932 cells expressing the Coxsackievirus A24‐reactive BCR.

Informed consent for the use of patient samples was obtained where required; in cases where consent was not applicable or could not be obtained, sample use was conducted in accordance with applicable ethical and regulatory provisions.

## 2 Discussion

Coxsackieviruses A and B are members of the *Enterovirus* genus and are common human pathogens, that can cause a variety of manifestations including skin, heart, and central nervous system disease. Whereas coxsackievirus B is one of the most common causes of aseptic meningitis in children under 2 years of age, coxsackievirus A has been most commonly associated with hand, foot, and mouth disease and herpangina [12]. Despite rarer than infections with group B, CNS involvement through coxsackievirus A has been reported [13]. Coxsackievirus A24 more specifically, was mostly associated with outbreaks of acute hemorrhagic conjunctivitis, but not with CNS involvement [14]. Rather rarely, coxsackievirus A24 has been detected in cases of acute flaccid paralysis [15]. However, these observations provide only limited evidence of neurotropism and cannot be regarded as evidence of CNS involvement.

In the observed case in our cohort, the antigen‐specific endpoint titer was eightfold lower in CSF than in plasma (1:50 vs. 1:400). Given the expected substantially lower total IgG concentration in CSF, this relatively preserved reactivity may suggest a disproportionate representation of Coxsackievirus A24‐reactive IgG in the CSF compartment. However, endpoint titers are semiquantitative, and an antigen‐specific antibody index incorporating paired total IgG concentrations and blood–CSF barrier function was not available. Therefore, these findings do not establish intrathecal antibody synthesis or CNS infection. They also do not allow us to distinguish recent or previous exposure from persistent or latent infection. Whether our findings reflect constant BCR activation through chronic or latent infection, or solely after exposure, remains unclear. No Coxsackievirus A24 RNA was detected in the available lymphoma tissue. This finding does not support the hypothesis of a persistent chronic or latent infection maintaining a stimulus of lymphomagenesis. However, tissue samples were stored for a considerably long period, which may have influenced the results. In addition, the clearest infectious association in PCNSL is observed in immunodeficiency‐associated disease, in which the lymphoma is frequently Epstein–Barr virus‐positive. In contrast, PCNSL in immunocompetent patients is generally EBV‐negative, and studies identifying other reproducible infectious triggers are scarce [16, 17].

Because enterovirus exposure is common, the isolated PCNSL reactivity could reflect previous infection, cross‐reactivity, or chance rather than disease‐associated enrichment. Published serological studies are difficult to compare because they were largely performed in outbreak‐related settings using whole‐virus neutralization assays. We therefore examined a geographically matched control cohort with the same recombinant antigen‐based ELISA. Reactivity was rare in controls and numerically more frequent in PCNSL (0.53% vs. 4.55%), but the presence of only one reactive individual in each cohort precludes firm conclusions and does not exclude a chance association.

Using previously described inducible BCR expression models, the Coxsackievirus A24‐derived antigen triggered phosphorylation of SYK, BTK, PLCγ2, and BLNK, together with increased c‐MYC expression, in cells expressing the corresponding PCNSL‐derived BCR. This antigen‐specific response supports the functional relevance of the interaction and, to our knowledge, represents the first demonstration that a viral antigen can activate a PCNSL‐derived BCR. However, as the experiments were performed in heterologous lymphoma cell lines, they demonstrate acute BCR signaling but do not establish persistent stimulation, enhanced lymphoma‐cell survival, or causality in PCNSL development.

The exploratory screening of a large number of viral constructs, the incomplete coverage of the custom‐made virome array, and the small cohort sizes limit conclusions regarding enrichment and disease specificity. In addition, the functional experiments were performed using recombinant BCR expression in ABC‐DLBCL cell lines. Although this model demonstrates antigen‐specific BCR signaling, it cannot reproduce the complete biological context of PCNSL. The retrospective design and rarity of the disease represent further limitations.

In conclusion, we identified and independently confirmed reactivity against a recombinant Coxsackievirus A24‐derived antigen in rare PCNSL samples. Functional experiments demonstrated that this antigen–BCR interaction can induce BCR pathway activation, strengthening its biological plausibility. These findings demonstrate antigen recognition but provide no evidence of viral persistence or a causal contribution to PCNSL development. Larger studies incorporating appropriate populations, molecular characterization of the recognized viral epitope, and functional assessment of cognate BCR signaling will be required to further determine the biological significance of this observation.

## Author Contributions

L.T. conceived the study. I.A.K. and L.T. designed and reviewed the experiments, interpreted the results, and wrote and revised the manuscript. N.F., E.R., F.F., and K.G. performed the experiments. F.F., K.G., S.H., F.S., and E.S. provided samples. O.C., K.D.P., M.B., and P.S. critically revised the manuscript. All authors reviewed and approved the final version of the manuscript.

## Funding

This study was supported by a grant of the Wilhelm Sander‐Foundation to Lorenz Thurner.

## Ethics Statement

This research was approved by the ethics committee of the Saarland Medical Board (Ärtztekammer des Saarlandes).

## Consent

This research was performed with patient consent, according to the recommendations of the Ethics Committee Saarland Medical Board.

## Conflicts of Interest

The authors declare no conflicts of interest.

## Supporting information




**Supporting Information**:jha270422‐sup‐0001‐SuppMat.docx.

## Data Availability

The data supporting the findings of this study are available from the corresponding author upon reasonable request.
