# Supplementary material for: Rare But Specific B‐Cell Receptor Reactivity Against Coxsackievirus A24 in Primary Central Nervous System Lymphomas
Source: EJHaem. 2026 Oct 8;7(5):e70422. doi: 10.1002/jha2.70422 (PMC13650266; doi:10.1002/jha2.70422)
Supplement: Supplementary file 1 [file JHA2-7-e70422-s001.docx]

# Supplementary Methods

## Recombinant B-cell receptors and VIROME protein microarray screening

Recombinant Fab fragments derived from the lymphoma B-cell receptors (BCRs) of 15 patients with primary central nervous system lymphoma (PCNSL) were screened at concentrations of 1, 10, and 20 µg/mL on a custom VIROME protein microarray representing 2,206 viral genes selected from 158 viruses. The array was generated using nucleic acid programmable protein array (NAPPA) technology at the Biodesign Institute, Arizona State University (ASU; Tempe, AZ, USA), where array screening and primary data analysis were performed. The Fab pool producing a reproducible signal against the Coxsackievirus A24-derived clone CaCD00959607 was subsequently deconvoluted by individual testing of its constituent Fabs. The candidate interaction was then assessed by dot blot and enzyme-linked immunosorbent assay (ELISA). A complete list of the viruses and constructs represented on the array is provided in Supplementary Table S1.

| **Table S1. Viruses and Proteins included in the screening array** |  |
| --- | --- |
| **Virus Name** | **Protein Number** |
| Human herpesvirus 4 | 89 |
| Human herpesvirus 5 | 165 |
| Human polyomavirus 1 | 5 |
| JC polyomavirus | 5 |
| Simian virus 40 | 6 |
| Human papillomavirus type 18 | 8 |
| Human papillomavirus type 51 | 8 |
| Human papillomavirus type 39 | 8 |
| Human papillomavirus type 45 | 8 |
| Human papillomavirus type 31 | 8 |
| Human papillomavirus type 35 | 8 |
| Human papillomavirus type 52 | 8 |
| Human papillomavirus type 58 | 8 |
| Human papillomavirus type 11 | 14 |
| Human papillomavirus type 33 | 12 |
| Human papillomavirus type 5 | 4 |
| Human papillomavirus type 6 | 5 |
| Human papillomavirus type 6b | 7 |
| Human papillomavirus type 8 | 3 |
| Human papillomavirus type 16 | 11 |
| Coxsackievirus B4 (strain E2) | 12 |
| Rubella virus (strain M33) | 6 |
| Mumps virus IA | 8 |
| Mumps virus | 3 |
| Human Endogenous Retrovirus IDDMK1,2-22 | 4 |
| rotavirus wa strain | 12 |
| Adenovirus | 16 |
| Hepatitis B virus | 10 |
| Chikungunya, strain Chik05115 | 9 |
| Semliki Forest virus, strain SFV | 9 |
| Sindbis virus, strain SIN | 9 |
| Varicella Zoster Virus | 94 |
| Vaccinia virus WR | 196 |
| Yellow fever vaccine, strain YF 17D | 11 |
| Measles, strain Ichinose WT | 5 |
| Measles (Schwarz vaccine) | 5 |
| Tioman virus | 3 |
| Enterovirus C | 13 |
| Human coronavirus 229E | 7 |
| Human coronavirus OC43 | 10 |
| Human herpesvirus 1 | 82 |
| Human mastadenovirus C strain human/USA/CL_42/1988/5[P5H5F5] | 32 |
| Human metapneumovirus | 10 |
| Human parainfluenza virus 1 | 12 |
| Human respiratory syncytial virus B | 11 |
| Influenza A virus (A/Puerto Rico/8/34/Mount Sinai(H1N1)) | 18 |
| Influenza A virus (A/Udorn/307/1972(H3N2)) | 17 |
| Primate bocavirus 1 | 4 |
| Rhinovirus B14 | 11 |
| Small anellovirus 1 | 3 |
| Human Parvovirus B19 | 6 |
| Dengue virus 1 | 6 |
| Dengue virus 2 | 6 |
| Dengue virus 3 | 9 |
| Dengue virus 4 | 9 |
| Onyong-nyong virus | 7 |
| Tick-borne encephalitis virus | 10 |
| zika virus | 9 |
| West Nile virus | 10 |
| Zaire ebolavirus | 7 |
| Sudan ebolavirus | 4 |
| Marburg marburgvirus | 5 |
| Bundibugyo virus | 6 |
| Human alphaherpesvirus 2 | 64 |
| Human betaherpesvirus 7 | 78 |
| Human adenovirus 41 | 31 |
| Human papillomavirus 9 | 7 |
| Adeno-associated virus - 2 | 10 |
| Human rotavirus C | 10 |
| Human respirovirus 3 | 7 |
| Human parainfluenza virus 4a | 6 |
| Human orthopneumovirus | 11 |
| Vesicular stomatitis Indiana virus | 4 |
| Rabies lyssavirus | 4 |
| Influenza C virus (C/Ann Arbor/1/50) | 9 |
| Human parechovirus 1 | 3 |
| Coxsackievirus A9 | 11 |
| Coxsackievirus B1 | 11 |
| Coxsackievirus B3 | 11 |
| Coxsackievirus B5 | 11 |
| Echovirus E11 | 11 |
| Coxsackievirus A24 | 11 |
| Hepatovirus A | 12 |
| Encephalomyocarditis virus | 11 |
| Coxsackievirus A16 | 2 |
| Human betaherpesvirus 6A | 78 |
| Human betaherpesvirus 6B | 92 |
| Coxsackievirus A2 | 9 |
| Echovirus E4 | 11 |
| California encephalitis virus | 3 |
| Enterovirus A71 strain Hau1 | 3 |
| Echovirus E30 | 3 |
| Hepatitis C virus genotype 1 | 11 |
| Coxsackievirus A10 | 11 |
| Coxsackievirus A22 | 11 |
| Coxsackievirus A4 | 3 |
| Coxsackievirus A5 | 3 |
| Coxsackievirus A8 | 3 |
| Enterovirus D68 | 11 |
| Echovirus E25 | 11 |
| Echovirus E7 | 3 |
| Echovirus E13 | 3 |
| Echovirus E14 | 3 |
| Echovirus E18 isolate NSW-V13A-2008-ECHO18 | 3 |
| Echovirus E21 | 11 |
| Echovirus E3 | 2 |
| GB virus C | 8 |
| Coxsackievirus B6 | 11 |
| Coxsackievirus B2 | 1 |
| Coxsackievirus A6 | 11 |
| Echovirus 9 strain Barty | 11 |
| Human mastadenovirus B | 36 |
| Norovirus GII | 8 |
| Human mastadenovirus A | 28 |
| Human mastadenovirus F | 29 |
| Human mastadenovirus D | 28 |
| Human picobirnavirus | 3 |
| Human coronavirus NL63 | 17 |
| Sapporo virus isolate Hu/GV/0003/Sollentuna/2016/SWE | 2 |
| Betapapillomavirus 4 | 6 |
| Human parechovirus 6 | 10 |
| Rhinovirus C | 11 |
| Influenza B virus (B/Lee/1940) | 5 |
| Human rhinovirus A1 | 9 |
| Human bocavirus 3 | 4 |
| Torque teno virus 3 | 5 |
| Torque teno virus 5 | 2 |
| Torque teno virus 7 | 2 |
| Torque teno virus 8 | 3 |
| Torque teno mini virus 1 | 2 |
| Torque teno midi virus 1 | 4 |
| Aichi virus 1 | 7 |
| Salivirus NG-J1 | 8 |
| Human bocavirus 2c PK | 4 |
| Human bocavirus 4 NI | 4 |
| Norovirus GI/Hu/JP/2007/GI.P3_GI.3/Shimizu/KK2866 | 7 |
| Parechovirus A | 9 |
| Human rubulavirus 2 | 6 |
| Human papillomavirus type 34 | 6 |
| Cowpox virus | 171 |
| Eastern equine encephalitis virus | 11 |
| Hepatitis E virus | 4 |
| Human gammaherpesvirus 8 | 76 |
| Human coronavirus HKU1 | 20 |
| Influenza A virus | 21 |
| H1N1 subtype | 8 |
| HIV-1 M:B | 17 |
| HIV-2 subtype A | 16 |
| H3N2 subtype | 15 |
| HIV-1 O_ANT70 | 13 |
| Adeno-associated virus - 8 | 3 |
| Adeno-associated virus - 9 | 2 |
| Adeno-associated virus - 7 | 3 |
| Adeno-associated virus - 1 | 3 |
| Adeno-associated virus - 5 | 3 |
| Human coronavirus OC43 | 22 |
| Middle East respiratory syndrome coronavirus | 21 |
| Severe acute respiratory syndrome coronavirus 2 (SARS-CoV-2) | 22 |
| Human coronavirus 229E | 19 |
| SARS coronavirus | 20 |
| Human coronavirus HKU1 | 18 |
| Human coronavirus NL63 | 20 |

## Generation of lymphoma cell lines expressing recombinant PCNSL-derived BCRs

## The paired heavy- and light-chain sequences of the Coxsackievirus A24-reactive PCNSL-derived BCR #1060 and the SAMD14/neurabin-I-reactive PCNSL-derived BCR #PCNSL7 were cloned into a modified doxycycline-inducible pRTS-1 vector comprising the heavy-chain variable and constant regions, transmembrane and cytoplasmic domains, a furin–2A sequence, and the light-chain variable and constant regions. TMD8 and U2932 cells were stably transfected with the corresponding constructs and selected with hygromycin. Recombinant BCR expression was induced with doxycycline concentration and verified by flow cytometry. Cells cultured without doxycycline served as uninduced controls.

## BCR pathway activation assay

BCR pathway activation was assessed by immunoblotting as described previously, with adaptation to the BCRs and antigens investigated in the present study. Following doxycycline induction, 1 × 10⁶ TMD8 or U2932 cells expressing BCR #1060 or BCR #PCNSL7 were incubated with no antigen, recombinant Coxsackievirus A24-derived antigen CaCD00959607, recombinant SAMD14, or anti-IgM as a positive control. Uninduced cells were analysed in parallel. CaCD00959607 and SAMD14 were used at 5 µg/mL, and anti-IgM at 1 µg/mL at 37°C.

Cell lysates were subjected to immunoblotting using antibodies against phosphorylated SYK (pTyr525/526; 1:2,000), PLCγ2 (pTyr759; 1:1,000), BTK (pTyr223; 1:1,000), and BLNK (pTyr96; 1:1,000) (B-Cell Signaling Sampler Kit, catalogue no. 9768; Cell Signaling Technology, Danvers, MA, USA). c-MYC was detected using a mouse monoclonal antibody at 1 µg/mL and actin was used as a loading control (1:2,000; A5060, Sigma-Aldrich). Following washing, membranes were incubated with peroxidase-conjugated anti-rabbit or anti-mouse secondary antibodies at 1:3,000. Signals were detected by chemiluminescence (Supplemental Figure 1 and 2)

Supplemental Figure 1 – Coxsackievirus A24-derived antigen induces BCR pathway activation in TMD8 and U2932 cells expressing recombinant BCR #1060

Supplemental Figure 2 - SAMD14 induces BCR pathway activation in TMD8 and U2932 cells expressing recombinant PCNSL7 BCR

**Cell proliferation assay**

Cell proliferation was assessed using the non-radioactive EZ4U assay (BI-5000; Biomedica, Vienna, Austria) according to the manufacturer’s instructions and as described previously, with adaptation to the BCRs and antigens used in this study. TMD8 and U2932 cells carrying doxycycline-inducible BCR #1060 or BCR #PCNSL7 were analysed in the presence and absence of doxycycline. Cells were seeded at 4 × 10⁴ cells/mL in 200 µL culture medium per well. Recombinant CaCD00959607 or SAMD14 was added at 1 µg/mL. Unstimulated cells served as negative controls, and anti-IgM at served as a positive control.

After incubation for 24 hours at 37°C, 20 µL of EZ4U chromogenic substrate was added to each well. Following incubation, formazan formation was measured at an optical density of 450 nm (Supplemental figure 3)

Supplemental Figure 3: Results of the cell proliferation assays with cell lines with recombinant BCR#1060 and BCR#PCNSL7

Cell proliferation was assessed using the EZ4U assay in cells expressing the doxycycline-inducible Coxsackievirus A24-reactive BCR #1060 or the SAMD14/neurabin-I-reactive PCNSL7 BCR. Cells were incubated with the Coxsackievirus A24-derived antigen Ca07, SAMD14, anti-IgM, or no antigen. Following doxycycline induction, Ca07 increased the proliferation signal in cells expressing BCR #1060, whereas SAMD14 increased proliferation in cells expressing the PCNSL7 BCR. Anti-IgM served as a positive control. Ø, unstimulated control.

# Supplementary References

1. Thurner L, Preuss K-D, Bewarder M, et al. Hyper-N-glycosylated SAMD14 and neurabin-I as driver autoantigens of primary central nervous system lymphoma. Blood. 2018;132(26):2744–2753. doi:10.1182/blood-2018-03-836932.

2. Thurner L, Fadle N, Regitz E, et al. B-cell receptor reactivity against Rothia mucilaginosa in nodular lymphocyte-predominant Hodgkin lymphoma. Haematologica. 2023;108(12):3347–3358. doi:10.3324/haematol.2023.282698.

3. Yu X, Bian X, Throop A, et al. Exploration of panviral proteome: high-throughput cloning and functional implications in virus-host interactions. Theranostics. 2014;4(8):808–822. doi:10.7150/thno.8255.
